# Comparative Study of Corneal Endothelial Cell Damage after Femtosecond Laser Assisted Deep Stromal Dissection

**DOI:** 10.1155/2014/731565

**Published:** 2014-07-10

**Authors:** Ting Liu, Jingjing Zhang, Dapeng Sun, Wenjie Sui, Yangyang Zhang, Dongfang Li, Zhaoli Chen, Hua Gao

**Affiliations:** State Key Laboratory Cultivation Base, Shandong Provincial Key Laboratory of Ophthalmology, Shandong Eye Institute, Shandong Academy of Medical Sciences, No. 5 Yan'erdao Road, Qingdao 266071, China

## Abstract

*Purpose.* To find a relatively safe designed stromal bed thickness to avoid endothelial damage for lamellar keratoplasty with an Allegretto Wavelight FS200 femtosecond laser. *Methods.* Twelve rabbits were randomly divided into 50 *μ*m and 150 *μ*m groups according to the anticipated residue stromal bed thickness preparation with a femtosecond laser. Six rabbits without laser cutting were used as a control group. Central endothelial images were analyzed with in vivo confocal microscopy and scanning electron microscopy. The apoptosis of endothelium was evaluated with Hoechst 33342 staining and a TUNEL assay. *Results.* The endothelium of the 50 *μ*m group had extensive injuries upon in vivo confocal and scanning electron microscopic observation, and minor injuries were observed in the 150 *μ*m group. Moreover, more apoptotic cells were observed in the 50 *μ*m group. *Conclusions.* When using a FS200 femtosecond laser assisted anterior lamellar keratoplasty, there was minor endothelium damage with a 150 *μ*m stromal bed, and a more than 150 *μ*m thickness stromal bed design may prevent the damage of corneal endothelium.

## 1. Introduction 

Penetrating keratoplasty has been widely used in patients with corneal blindness [1, 2]. But the risk of immune rejection and postoperative astigmatism following penetrating keratoplasty still affect postoperative prognosis [3]. With the deepening understanding of the anatomical structure of the cornea and the development of modern microsurgical techniques, an increasing number of ophthalmologists tend to use split keratoplasty, which only replaces the diseased part of cornea. So, anterior lamellar keratoplasty was used to treat corneal stromal lesions without apparent endothelial cell damage, and lamellar posterior keratoplasty was applied in patients with endothelial decompensation. During the split keratoplasty procedure, the two primary concerns are cutting the diseased stroma precisely and protecting the host's endothelium [4, 5].

The femtosecond laser was a modern transition medical instrument, and it presented remarkable prospects in various medical fields. In the field of ophthalmology, femtosecond lasers provide advantages for the modern corneal surgeon and have been rapidly adopted to create corneal flaps of predetermined depth during LASIK. The femtosecond laser is now capable of dissecting a thin, of uniform thickness, lamellar disk of cornea not only to be used for lamellar posterior but also to be used for anterior lamellar keratoplasty [6, 7]. With the use of proprietary software, a femtosecond laser can create a better graft-host fit with less postoperative astigmatism and can increase graft-host interface surface area and fit. However, femtosecond lasers are long wavelength lasers, and a light burst will damage the surrounding corneal tissue [8, 9]. Corneal endothelial cells comprise one of the most important layers of the corneal cells and cannot be regenerated when injury results in corneal endothelial cell decompensation. During the femtosecond laser cutting, the laser energy was generated for photodisruption of tissue, which may be absorbed by the cornea tissue and damage the nearby corneal endothelial cells. Therefore, there are still some concerns about the use of a femtosecond laser to create a <100 *μ*m thick stromal bed, in which the endothelium may be damaged when cutting close to the delicate cells.

Although the femtosecond laser was a delicate and accurate machine in cutting, there still existed deviation during the cutting procedure. The predictability of the cutting was an important factor to consider when we need a femtosecond laser assisted deep stromal dissection. In the present study, we used FS200 femtosecond laser to make designed 50 *μ*m and 150 *μ*m recipient stromal bed and observe its effects on the corneal endothelium. Our aim was to find a relatively safe designed stromal bed thickness to avoid endothelial damage for lamellar keratoplasty with an Allegretto Wavelight FS200 femtosecond laser.

## 2. Materials and Methods

### 2.1. Animals

All animal experiments were carried out in accordance with The Chinese Ministry of Science and Technology Guidelines on the Humane Treatment of Laboratory Animals (vGKFCZ-2006-398) and the Association for Research in Vision and Ophthalmology (ARVO) Statement for the Use of Animals in Ophthalmic and Vision Research. This study was approved by the Animal Care and Use Committee on the Ethics of the Shandong Eye Institute. Eighteen male or female New Zealand White rabbits (2–2.5 kg) were used in this study. Rabbits were anaesthetized with a combination of ketamine and xylazine (ketamine 40 mg/kg, xylazine 20 mg/kg; IM). Proparacaine drops were used for topical anesthesia during surgery. Animals were sacrificed with an overdose intravenous injection of pentobarbital.

### 2.2. Groups

In accordance with planting bed thickness, the animals were subdivided into three groups: a residual bed thickness of 50 *μ*m or 150 *μ*m and a control group, with 6 rabbits in each group. The right eye of each rabbit was used, and central corneal thickness was measured with anterior segment ocular coherence tomography (AS-OCT) before surgery.

### 2.3. Femtosecond Laser Cutting Procedure

Lamellar cutting was performed using an Allegretto Wavelight FS200 femtosecond laser (Wavelight AG, Erlangen, Germany). A lamellar keratoplasty cut configuration was chosen for the graft with diameters of 8.5 mm and an angle of 90°. ASOCT was used to help guide the desired thickness of the graft. The femtosecond laser settings included a bed cut energy of 1.0 *μ*J and a side cut energy of 0.1 *μ*J. The spot separation at the bed cut was 6.0 *μ*m, and the line separation was 6.0 *μ*m. The spot/line separation of the bed cut was 10.0/10.0 *μ*m. The surgical procedure was as follows: the speculum was placed adjacent to the rabbit's eye, the eyelid was opened, and the suction ring was positioned on the right eye. After the suction ring had an appropriate vacuum pressure (500 mmHg), the applanation cone was guided into the suction ring using the laser joystick, and the laser pedal was pressed [10]. In the rabbits in control group, suction rings were also put on the right eye and vacuum pressure was added, but the laser pedal was not pressed.

### 2.4. Slit-Lamp Photography and Optical Coherence Tomography (OCT) of Rabbit Corneas

Before the animals were sacrificed, slit-lamp photographs (Nikon FS-3V; Nikon, Tokyo, Japan) were captured. Corneal cross-sectional visualization in the rabbits was performed using a Visante ASOCT unit (Carl Zeiss Meditec) 12 hours after surgery.

### 2.5. In Vivo Scanning Confocal Microscopy of Rabbit Corneas

Confocal microscopy images were obtained before surgery and 12 hours after surgery. Before the examination, each rabbit was anaesthetized as described above. A lid speculum was placed to separate the eyelids of the right eye of each rabbit. After one drop of 0.5% proparacaine hydrochloride (Alcon Laboratories, Fort Worth, Texas, USA) was applied, the central cornea was examined with a scanning confocal microscope (Heidelberg HRT III, German). The center and four quadrant points within 6 mm of the central cornea were examined. Approximately 100 sequential images were obtained from the endothelium to the epithelium during a single examination.

### 2.6. Evaluation of the Corneal Endothelium

Endothelial cell damage was assessed in the right eyes of rabbits, and corneal buttons were excised for endothelial staining and electron microscopy after 12 hours. The corneas were collected, and divided equally into 3 parts. One-third of the cornea was examined by alizarin red and Hoechst staining (Beyotime Institute of Biotechnology, Shanghai, China). The other two parts were used for scanning electron microscopy and histopathology assay. Briefly, 1% alizarin red was added to the cornea, which was incubated for 2 minutes at room temperature and washed three times in normal saline. Next, Hoechst 33342 stain was added to the cornea and incubated for 15–20 minutes at 4°C in the dark. Endothelial cells were observed using a Fluorescence E800 microscope (Nikon, Tokyo, Japan) [11]. Hoechst 33342 staining showed that the nucleus of apoptotic cells was uneven and shrinking. Six microscopy fields (400x) were randomly chosen in each cornea of three groups, and average apoptosis positive cells per high power field were counted for statistical analysis.

### 2.7. Scanning Electron Microscopy (SEM)

The corneal buttons were half cut and fixed in 4% glutaraldehyde in 0.05 M cacodylate buffer for 1 h, washed in a buffered solution of 0.2% sucrose-cacodyl for 4−10 h, postfixed in 1% osmium tetroxide in veronal acetate buffer for 1 h, and dehydrated through a series of ethanol. The samples were then dried and mounted on SEM stubs using carbon adhesive tabs. They were then sputter-coated with a 10 nm thick layer of gold (Bal-Tec) and examined with a scanning electron microscope (JSM-840; JEOL, Tokyo, Japan).

### 2.8. Histopathology and TUNEL Assay

The corneal buttons were half cut along the central line. Serially graded ethanol baths followed by xylene were used to dehydrate the tissues before they were immersed in paraffin wax. The samples were embedded in paraffin molds, sectioned at 4 *μ*m thickness, and mounted on glass slides. A hematoxylin-eosin stain was used for microscopic examination and evaluation.

Free 3′-OH DNA ends were detected in situ by the terminal deoxyribonucleotidyl transferase-mediated (TUNEL) labeling method according to the manufacturer's instructions using the in situ cell death detection kit, POD (Roche, Mannheim, Germany). Briefly, the sections were incubated with terminal deoxynucleotidyl transferase (TDT) and a nucleotide mixture in a reaction buffer and then incubated with an anti-FITC antibody conjugated with horseradish peroxidase. Peroxidase activity was detected by exposure of the sections to 3-amino-9-ethylcarbazole (AEC) solution (Maxin, Fujian, China), which were finally counterstained with hematoxylin. For negative controls, the nucleotide mixture was used instead of the TDT enzyme solution. Positive cells stained red in high-magnification fields were counted [12]. Six high power fields (400x) were randomly chosen in each slide of three groups, and average positive cells were counted per high power field for statistical analysis.

### 2.9. Statistical Analysis

Significant differences between endothelium counts and apoptotic cell counts among the three groups were evaluated with the Student-Newman-Keuls one-way ANOVA using SPSS 17.0 software. The mean ± standard deviation is shown, and *P* values < 0.05 were considered to be statistically significant.

## 3. Results

### 3.1. Femtosecond Laser Cutting Accuracy

**Figure 1:**
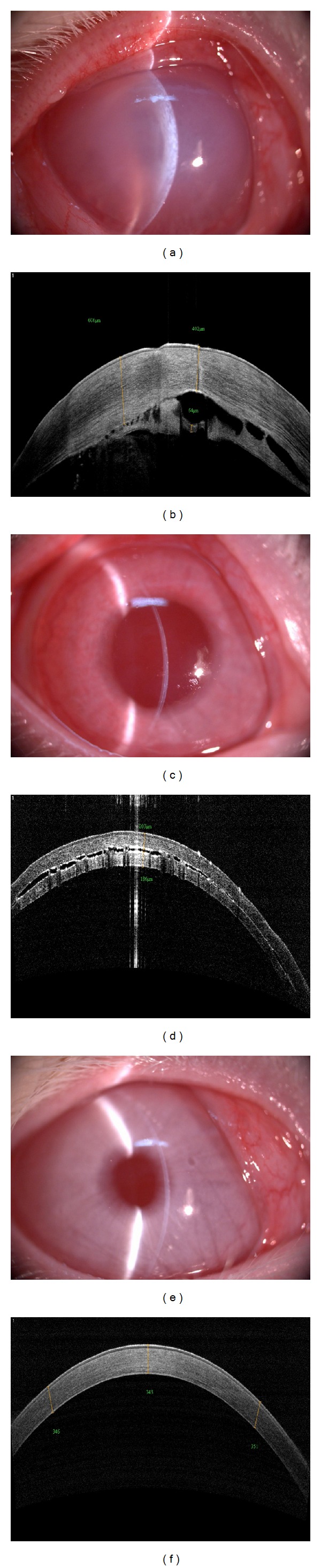
Rabbit slit-lamp microscopic examination and OCT images after femtosecond laser cutting. (a) The obvious corneal edema can be observed. (b) The irregular interlamellar space can be seen in the 50 *μ*m group and connected to the anterior chamber. (c) No obvious corneal edema can be observed. (d) There was a relatively regular interlamellar space in the 150 *μ*m group. (e) A clear cornea can be observed. (f) No interlamellar space was present in the control group.

The femtosecond laser is a near infrared laser, and it causes photodisruption of tissue. The generation and confluence of plasma cavitations in a plane will result in tissue dissection. After the femtosecond laser cutting, corneal edema occurred in the 50 *μ*m group. The corneas of two rabbits in the 50 *μ*m group were unexpectedly penetrated by the laser, and obvious corneal edema still could be observed after 12 hours. The irregular interlamellar space could be seen in the 50 *μ*m group and was connected to the anterior chamber. In the 150 *μ*m group, there were regular shallow interlamellar space in the cornea and no obvious edema could be observed after 12 hours. In the control group, the corneas of rabbits were clear throughout the experiment (Figure 1).

No differences were found in the corneal thickness before surgery. The corneal thicknesses of three groups were 351.3 ± 12.9 *μ*m (50 *μ*m group), 345.7 ± 11.9 *μ*m (150 *μ*m group), and 353.2 ± 12.1 *μ*m (control group), respectively. The cutting thickness was calculated by subtracting the anticipated stromal bed thickness. After laser cutting, the achieved residual bed thickness was 40.0 ± 34.8 *μ*m (range: 0–77 *μ*m) in the 50 *μ*m group and 175.2 ± 6.3 *μ*m (range: 167–185 *μ*m) in the 150 *μ*m group.

The expected cutting depth is 301.2 ± 13.0 *μ*m (range: 293–327 *μ*m) for 50 *μ*m group, 195.7 ± 11.8 *μ*m (range: 179–215 *μ*m) for 150 *μ*m group, and 0 *μ*m for control group.

The average deviation from expected target results was 30.0 ± 14.6 *μ*m (range: 14–50 *μ*m) in the 50 *μ*m group and 25.2 ± 6.3 *μ*m (range: 17–35 *μ*m) in the 150 *μ*m group, and there was no significant difference (*P* = 0.413).

### 3.2. Evaluation of the Endothelial Cell Density In Vivo

**Figure 2:**
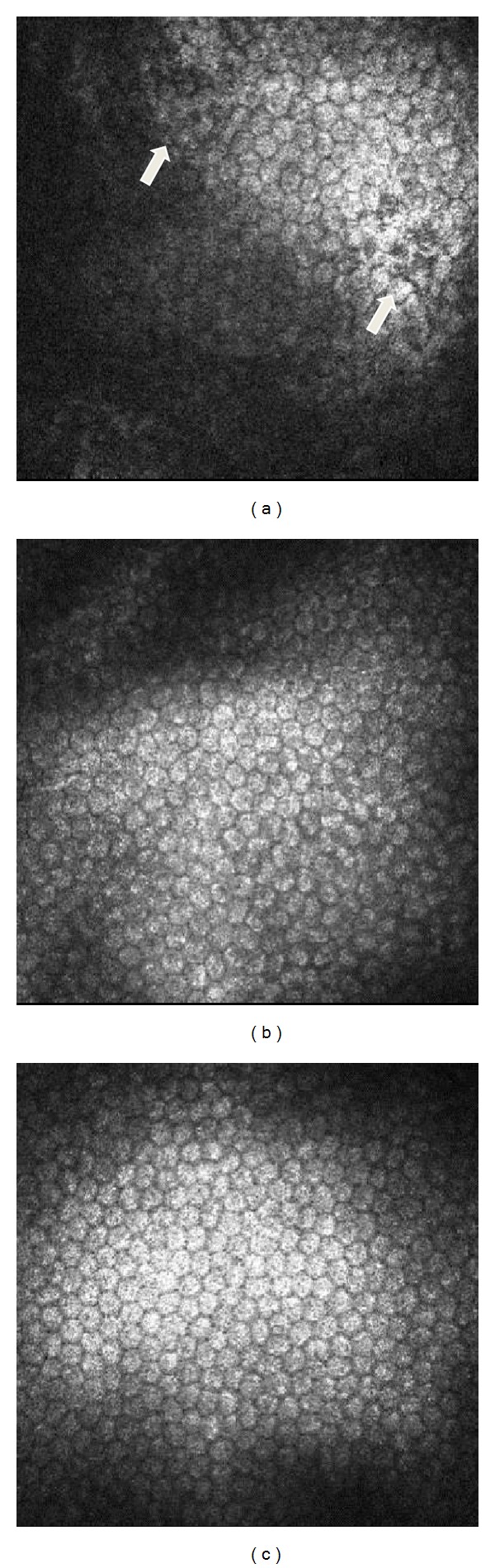
Representative images of the endothelium from in vivo confocal microscopy after femtosecond laser cutting. (a) Many lost hexagonal endothelium cells could be observed in the 50 *μ*m group. ((b) and (c)) The endothelium had a regular hexagonal structure in the 150 *μ*m group and the control group.

There were no differences in the endothelium cells density between each group before surgery. After the laser cutting, there were many lost hexagonal endothelium cells in the 50 *μ*m group, as observed with in vivo confocal microscopy, which was not found in the 150 *μ*m group or the control group (Figure 2). The endothelium count of 1691.3 ± 277.9 cells/mm^2^ in the 50 *μ*m group was significantly poorer when compared with 2797.5 ± 238.1 cells/mm^2^ in the 150 *μ*m group and 2912 ± 273.1 cells/mm^2^ in the control group (*P* = 0.000 and *P* = 0.000, resp.).

### 3.3. Evaluation of Corneal Endothelium Apoptosis

**Figure 3:**
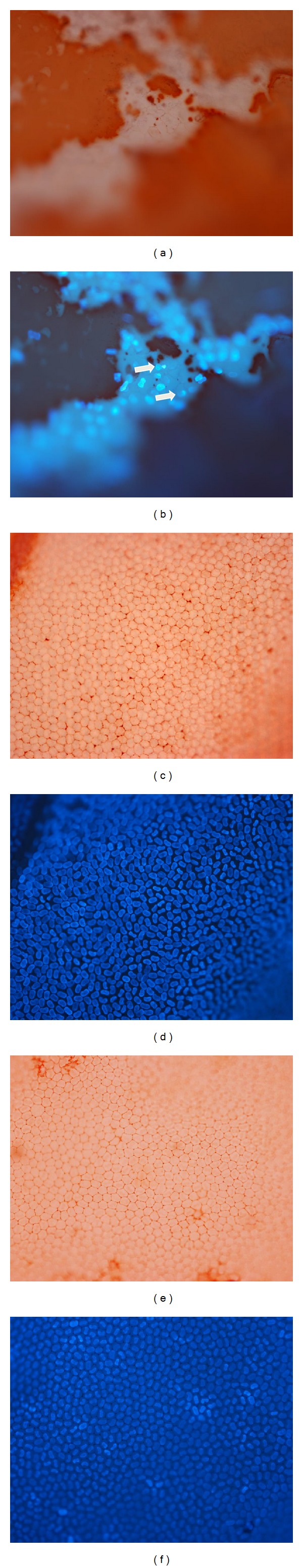
Alizarin Red and Hoechst 33342 staining of the endothelium after femtosecond laser cutting. (a) The endothelial cell borders of corneal buttons in the 50 *μ*m group were not clear, and in some place the endothelial cells were completely lost. (b) Hoechst 33342 staining showed that the nucleus was uneven and shrinking, with many apoptotic cells present in the 50 *μ*m group. ((c) and (e)) The endothelial cell borders of the 150 *μ*m group and the control group were clear and had a normal hexagonal structure. ((d) and (f)) No apoptotic cells could be detected in the 150 *μ*m group or the control group.

Apoptosis was a main mechanism involved in corneal endothelium loss. The endothelium decreased with aging, and less than 500 cells/mm^2^ in human will induce the endothelium decompensation. With alizarin red staining, the endothelial cell borders of the corneal buttons in the 50 *μ*m group were not clear, and in some places the endothelial cells were completely lost (Figure 3(a)). The endothelial cell borders of the 150 *μ*m group and control group were clear and maintained a hexagonal structure (Figures 3(c) and 3(e)). Hoechst staining showed that the nucleus was uneven and shrinking, with many apoptotic cells present in the 50 *μ*m group (Figure 3(b)). No apoptotic cells could be detected in the 150 *μ*m group or the control group (Figures 3(d) and 3(f)).

### 3.4. Scanning Electron Microscopy (SEM)

**Figure 4:**
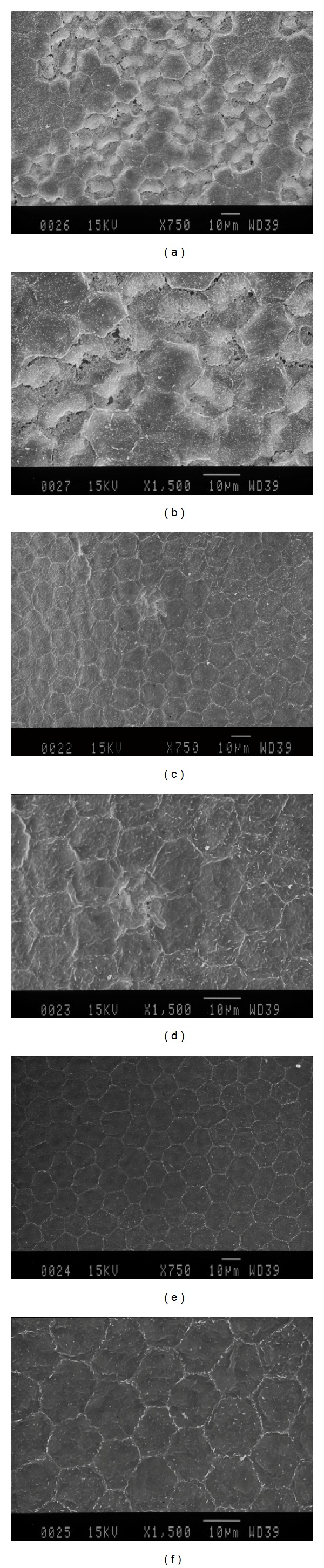
Representative images from scanning electron microscopy (SEM) of endothelial cells in the cutting area after femtosecond laser cutting. ((a) and (b)) The laser created a substantial endothelial damage area in the 50 *μ*m group. The damaged endothelium presented with many small cavities resembling holes in a sponge, and the normal hexagonal structure was destroyed. ((c) and (d)) Only sporadic cells presented with swollen changes in the 150 *μ*m group. ((e) and (f)) No obvious damage was noted in the control group.

SEM images of endothelial cells were analyzed in the cutting area. The laser created substantial endothelial damage areas in the 50 *μ*m group (Figures 4(a) and 4(b)). The damaged endothelium presented with many small cavities resembling holes in a sponge, and the normal hexagonal structure was destroyed. Only sporadic cells presented with swollen changes in the 150 *μ*m group (Figures 4(c) and 4(d)), and no obvious damage was noted in the control group (Figures 4(e) and 4(f)).

### 3.5. Histological Evaluation and TUNEL Assay

**Figure 5:**
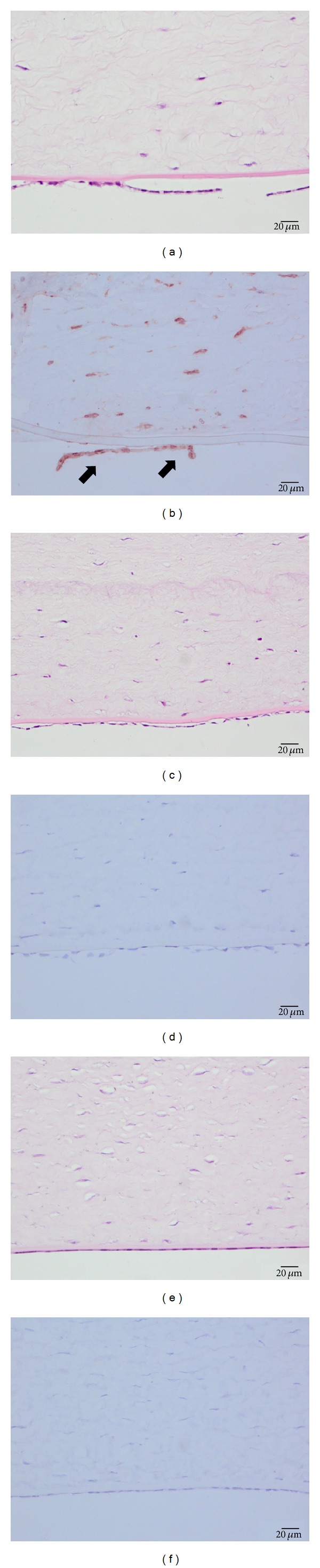
Histological evaluation and TUNEL assay of endothelial cells after femtosecond laser cutting. (a) The corneal stroma was edematous and only a small number of keratocytes and endothelial cells could be found in the 50 *μ*m group. (c) Most of the deep stromal cells and the endothelium of the 150 *μ*m group were intact and only swollen changes could be seen in the 150 *μ*m group. (e) No endothelium damage or swollen changes could be observed in the control group. (b) There were many TUNEL positive cells in the 50 *μ*m group. ((d) and (f)) No TUNEL positive cells were found in the 150 *μ*m group or the control group.

In the 50 *μ*m group, the corneal stroma was edematous and only a small number of keratocytes and endothelial cells could be found (Figure 5(a)). Most of the deep stromal cells and endothelial cells of the 150 *μ*m group were intact, and only swollen changes could be seen in the 150 *μ*m group (Figure 5(c)). No endothelium damage or swollen changes could be observed in the control group (Figure 5(e)). There were many TUNEL positive endothelium cells and keratocytes in the 50 *μ*m group (Figure 5(b)), but no TUNEL positive endothelium cells were found in the 150 *μ*m group or the control group (Figures 5(d) and 5(f)).

### 3.6. Statistical Evaluation of Endothelial Cells and Apoptosis

**Figure 6:**
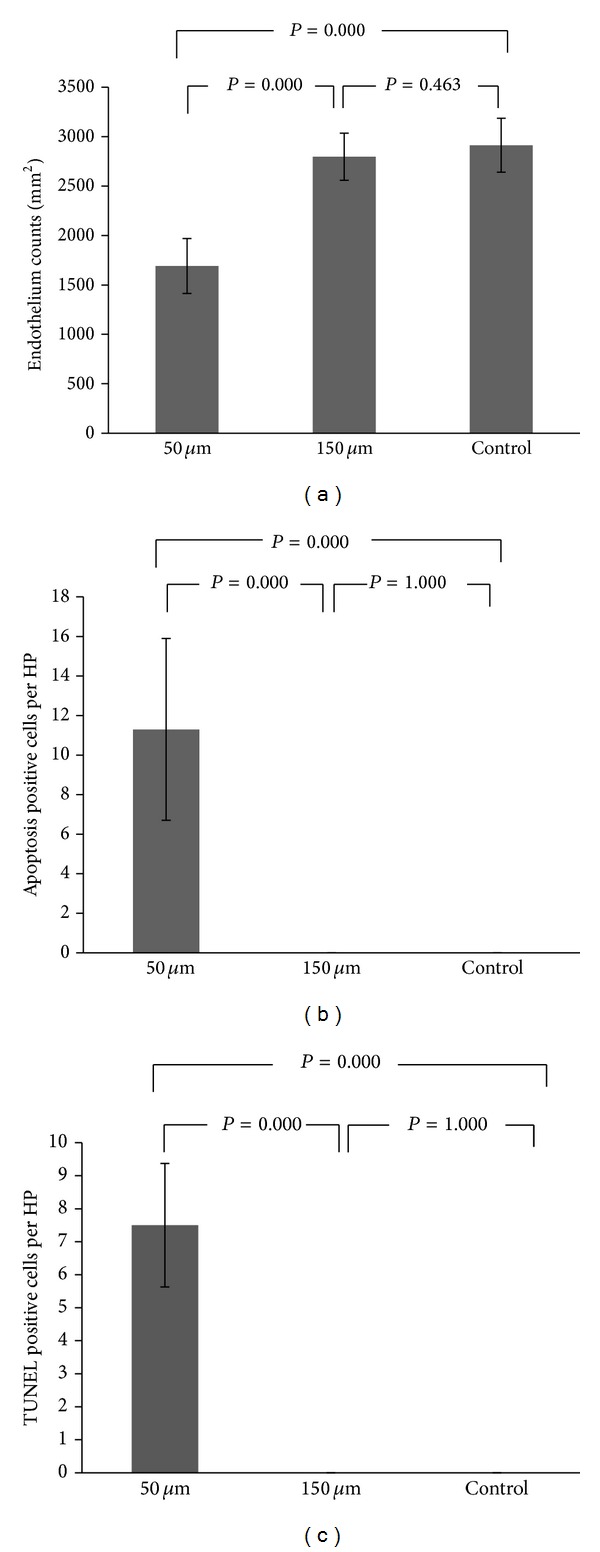
Evaluation of endothelial cells and apoptosis changes after femtosecond laser cutting. Significant differences were found between the 50 *μ*m group and the 150 *μ*m group (*P* < 0.05) including (a) endothelial cell count, (b) the number of apoptotic cells per high power field, and (c) the number of TUNEL positive cells per high power field.

Significant differences were found between the 50 *μ*m group and the 150 *μ*m group (*P* < 0.05) in the endothelial cell counts, the number of apoptotic cells per area as viewed in high magnification, and the number of TUNEL positive cells per area as viewed in high magnification (Figure 6).

## 4. Discussion

In recent years, split keratoplasties, such as anterior lamellar keratoplasty (LKP) and lamellar posterior keratoplasty (Descemet's stripping automated endothelial keratoplasty), have been used more frequently to replace diseased cornea [13, 14]. Traditionally, the stromal bed or the endothelium grafts were prepared by manual sectioning, and the qualities of the stromal bed were determined by the skills of the surgeon. The use of femtosecond lasers to create a stromal bed had proved to be a viable method, because the lasers can accurately cut the full thickness of the cornea and effectively avoid the possible iatrogenic aberrations associated with microkeratomes [15–17]. Analysis of femtosecond-dissected donor tissues using atomic force microscopy images on a submicron scale proved that the surface quality of posterior cornea is significantly improved when compared with that provided by mechanical microkeratomes [18]. The easy and fast preparation of the stromal bed using femtosecond laser cutting was a remarkable development for lamellar keratoplasty [19, 20].

However, during femtosecond laser cutting, the deposited energy may damage nearby corneal tissue. It has been reported that keratocyte apoptosis and inflammation could occur after femtosecond laser cutting in refractive surgery [21]. If the cutting injury was severe, the endothelium could be damaged during deep lamellar keratoplasty. The effect of femtosecond laser on corneal endothelial health was a main concern when using the femtosecond laser to make a deep lamellar cutting [22–25]. The residual stromal bed may act as a cushion to protect the endothelium from the damage of deposited energy. For anterior lamellar keratoplasty, if the deep corneal stroma was affected, we may need to create a thinner stromal bed. However, making a thin stromal bed will increase the risk of endothelial damage. Until now, we lacked an accurate study to determine whether the anticipated cutting thickness of the FS200 femtosecond laser was stable and what thickness of a residual stromal bed may be safe for lamellar keratoplasty during femtosecond cutting. In the present study, we provide comparative evidence to determine the accuracy of femtosecond laser cutting and the safety of different cutting thickness as we make a more precise stromal bed for deep lamellar keratoplasty.

The accuracy of femtosecond laser cutting depth is very important for guaranteeing the safety of operations. When cutting at 420 *μ*m to 500 *μ*m thickness using a low-pulse energy, high-frequency (LPEHF) femtosecond laser (Ziemer Femto LDV; Ziemer Ophthalmic Systems, Port, Switzerland), Phillips et al. reported that the cutting accuracy was 17 to 54 *μ*m [26]. In our study, when cutting at 293 to 327 *μ*m thickness using the average deviation of the achieved residual bed thickness from the expected target thickness, it did not reach statistical significance, which proved the acceptable cutting ability of FS200 femtosecond laser.

When using a LDV femtosecond laser (energy < 100 nJ) to make a tissue thickness of approximately 70 *μ*m, Phillips et al. reported that there was no endothelial cell damage difference between experimental and control corneas. However, Kimakura et al. [27] reported that the mean ratio of damaged corneal endothelial cells in the group with a remaining depth of 70 *μ*m was significantly higher than that in the group with a remaining depth of 150 *μ*m. The reason may be they used a 150 kHz femtosecond laser (energy 1.50 mJ). In a previous report, when cutting at a greater depth, the irregular stroma could have been caused by the increased scatter and attenuation of laser efficacy [28]. In our study, we could detect more endothelial cell damage and irregular cutting interface in the 50 *μ*m group, but no obvious endothelial cell damage changes and a smooth interface could be found in the 150 *μ*m group. One reason for this result may be more cutting thickness and the thinner stroma left in the 50 *μ*m group; another reason may be more energy exported with the FS200 femtosecond laser (1.0 *μ*J) than that of LDV femtosecond laser (<100 nJ) during the cutting.

Clinically, a less thick bed and smooth interface play an important role to ensure quality of vision after surgery. The corneal endothelial damage was minor and smooth interface could be found when we prepared 150 *μ*m thickness bed. But perforation occurred in the 50 *μ*m bed rabbits, and more endothelial cells were damaged. This proved that at present femtosecond laser assisted less thick bed preparation may lack safety. A much thicker corneal bed is often difficult to excise lesion, and clinical applications are limited. Therefore, from the viewpoint of safety and clinical application, leaving 150 *μ*m bed may be ideal. But our study involved a limited number of animals, and a larger study or human samples may be needed to further prove the safe stromal bed thickness with femtosecond laser cutting. We used an Allegretto Wavelight FS200 femtosecond laser, and the results may not be applicable for other types of femtosecond laser.

In conclusion, when using a FS200 femtosecond laser assisted anterior lamellar keratoplasty, there was minor endothelium damage with a 150 *μ*m stromal bed, and a more than 150 *μ*m thickness stromal bed design may prevent the damage of corneal endothelium.
